# Prospective study investigating hypofractionated proton beam therapy in patients with inoperable early stage non-small cell lung cancer

**DOI:** 10.3389/fonc.2024.1296172

**Published:** 2024-02-20

**Authors:** Kyungmi Yang, Jae Myoung Noh, Hye Yun Park, Hongseok Yoo, Sun Hye Shin, Hongryull Pyo

**Affiliations:** ^1^ Department of Radiation Oncology, Samsung Medical Center, Sungkyunkwan University School of Medicine, Seoul, Republic of Korea; ^2^ Division of Pulmonary and Critical Care Medicine, Department of Medicine, Samsung Medical Center, Sungkyunkwan University School of Medicine, Seoul, Republic of Korea

**Keywords:** non-small cell lung cancer, radiotherapy, proton therapy, hypofractionated radiotherapy, quality of life

## Abstract

**Purpose:**

To report the results of hypofractionated proton beam therapy (PBT) for the treatment of early stage lung cancer in patients not suitable for surgical resection.

**Methods:**

Data from 27 adult patients, who were diagnosed with inoperable cT1-3N0 non-small cell lung cancer (NSCLC) between March 2018 and August 2020, were analyzed. PBT was prescribed as 64 Cobalt Grey equivalents delivered in 8 fractions (Sumitomo, Japan). The primary endpoint was local control; secondary endpoints included overall survival, quality of life, and grade ≥3 toxicity.

**Results:**

The median follow-up was 28.9 months (range, 1.1–62.1 months). During follow-up, 13 (48.1%) patients experienced disease progression, including local progression in 7. Two-year local control rates were 73.5%, 85.7% for T1, and 61.4% for T2-3. The worse local control rate was observed in those with large clinical target volumes (≥ 47.5 cc) and heavy smoking history (≥30 pack-years). The two-year overall survival rate was 76.5%. Grade 3 radiation-related toxicities were observed in 2 (7.4%) patients. In the European Organization for Research and Treatment of Cancer Quality of Life Core 30 results, the global score did not change significantly from baseline. However, dyspnea score increased from 19.8 before PBT to 33.3 at 4 months’ post-PBT (p=0.047) and was maintained until 13 months (p=0.028).

**Conclusion:**

Hypofractionated PBT was a safe treatment option for inoperable early stage NSCLC and appeared to be appropriate for small tumor volumes. However, local control for larger tumors requires further improvement.

## Introduction

Surgical resection is the treatment of choice for patients with early stage non-small cell lung cancer (NSCLC) (1, 2). For medically inoperable patients of advanced age, poor performance status, impaired cardiopulmonary function, and comorbidities, definitive radiation therapy (RT) is recommended as an alternative to surgical resection. Although conventionally fractionated RT is known to yield unsatisfactory clinical outcomes, hypofractionated RT is believed to enhance local control (3–5). Stereotactic body radiotherapy (SBRT), an extreme type of hypofractionation, can yield excellent local control; however, it has limitations in terms of tumor location and size associated with severe complications (6, 7). Therefore, smaller hypofractionation sizes using 6–15 fractions are recommended for patients deemed to be at high risk for SBRT (8–10).

Proton beam therapy (PBT), which has a unique depth-dose curve with a Bragg peak, decreases the dose to at-risk surrounding organs (11). This feature could be beneficial in patients with poor pulmonary function with or without underlying lung disease(s); however, the clinical benefit of PBT versus photon SBRT is unclear, especially for small peripheral lesions (12, 13). Several studies have examined the feasibility of hypofractionated PBT for early stage NSCLC, including centrally located lesions, and excellent local control (86%–97%) and low grade 3 pulmonary toxicity (0–3.6%) have been reported (14–16).

Based on this background, this prospective study aimed to investigate the utility of hypofractionated PBT at initiation of treatment for inoperable early stage NSCLC and to report the clinical outcomes.

## Methods

### Study design and patients

This study was approved by the Institutional Review Board of Samsung Medical Center (Seoul, South Korea; 2017-10-012). All participants provided informed written consent. Eligible patients were > 20 years of age, with pathology-confirmed or radiologically diagnosed NSCLC that was not suitable for surgical resection determined by multidisciplinary review, cT1-3N0 according to the 8th American Joint Committee on Cancer (AJCC) Cancer Staging Manual, forced expiratory volume in 1 s ≥ 1 L, and an Eastern Cooperative Oncology Group (i.e., “ECOG”) performance status of 0 to 2. For safety reasons, this study focused only on patients with tumors located at least 2 cm away from the proximal bronchial tree, 1 cm away from the esophagus, and at least 2 cm from major mediastinal structures such as the heart and great vessels. Patients with recurrence of metastatic disease, a history of previous RT to the chest, or a history of another malignancy within 2 years were excluded.

### Procedures

Pre-treatment assessments consisted of medical history and physical examination, Charlson Comorbidity Index, laboratory investigations, pulmonary function test(s) (PFT), and quality of life (QoL) questionnaires. Clinical staging, according to the 8th AJCC Cancer Staging Manual, was based on chest computed tomography (CT) and ^18^fluoro-deoxyglucose (FDG) positron emission tomography (PET) scans.

For treatment planning, four-dimensional simulation CT scans performed using a thickness of 2.5 mm were acquired. When tumor motion was < 1 cm, the internal target volume (ITV) was delineated by combining all gross tumor volumes (GTV) in each respiratory phase, and a 5 mm margin from the ITV was added to generate the clinical target volume (CTV). The planning target volume was delineated by adding a 5 mm margin to the CTV. Breath-hold or respiratory gating was considered for patients with tumor motion ≥ 1 cm. The prescribed dose was 64 Gy delivered in 8 fractions. RayStation (RaySearch Laboratories, Stockholm, Sweden) was used for treatment planning, and a fixed value of 1.1 was considered for the relative biological effectiveness of PBT. Passive scattering with wobbling or continuous line scanning was performed using a proton therapy system at the authors’ institution (Sumitomo, Niihama, Japan) (17). Daily image guidance was performed before each treatment session using cone-beam CT (VeriSuite, MedCom, Darmstadt, Germany).

### Assessments and statistical analysis

Patients were assessed 1 and 4 months after the completion of PBT, and then every 3 months for 2 years thereafter. Chest CT and PFTs were performed at every visit, and FDG-PET was performed 4 months after completion of PBT. The QoL questionnaire, based on the European Organization for Research and Treatment of Cancer Quality of Life Core 30 (EORTC-QLQ-C30), was completed at 1, 4, and 13 months after completion of PBT.

The primary endpoint of this study was local control rate, while secondary endpoints included overall survival (OS) rate, changes in QoL, and grade ≥ 3 toxicity according to the Common Terminology Criteria for Adverse Events, version 4.03. Sample size calculation was based on the assumption that, given a two-sided α-value of 0.05 and power of 0.85, the two-year local control rate would be 60% and 85% after conventional photon treatment and PBT, respectively. An accrual rate of 3 patients per month was assumed and an additional follow-up period of 2 years. Therefore, the required sample size was 24 patients. Considering a dropout rate of 10%, the total planned sample size was 27.

OS was calculated using the Kaplan–Meier method from the start date of PBT to the date of death at the last follow-up and compared between subgroups using the log-rank test. Periodic changes in lung function and QoL from baseline were compared using a paired *t*-test. Differences with p<0.05 were considered to be statistically significant. Statistical analyses were performed using SPSS version 27.0 (IBM Corporation, Armonk, NY, USA).

## Results

### Patient characteristics

A total of 27 patients (median age, 74 years; range, 56–84 years; 92.6% male) were included in the analysis. A review of smoking status revealed a median of 40 pack-years, with 70.4% of patients having a smoking history ≥ 30 pack-years. Underlying lung disease(s) was prevalent in two-thirds of the patients, with chronic obstructive pulmonary disease, idiopathic pulmonary fibrosis, and combined pulmonary fibrosis and emphysema accounting for 25.9%, 25.9%, and 14.8% of cases, respectively. Tumor stage revealed distributions of 51.9%, 33.3%, and 14.8% for stages T1, T2, and T3, respectively. Histologically, the majority of patients had adenocarcinoma (37.0%), followed by squamous cell carcinoma (22.2%), and cases in which histology was not confirmed (40.7%). The median CTV was 53.4 cc. Patients were categorized based on the breath control method used during treatment; 63.0% used free breathing, while 37.0% used respiratory gating or breath-hold techniques (**Table 1**). The dose–volume parameters in the lungs, heart, esophagus, and spinal cord are provided in **Supplementary Table 1**.

**Table 1:** Patient characteristics.

Characteristics	N (%)
Age, years (range) < 75 ≥ 75	Median 74 (56 – 84) 14 (51.9%) 13 (48.1%)
Sex Female Male	2 (7.4%) 25 (92.6%)
Smoking, pack-year (range) < 30 ≥ 30	Median 40 (0 – 100) 8 (29.6%) 19 (70.4%)
Underlying lung disease Non-specific COPD IPF CPFE	9 (33.3%) 7 (25.9%) 7 (25.9%) 4 (14.8%)
Clinical T-stage T1 T2 T3	14 (51.9%) 9 (33.3%) 4 (14.8%)
Histology Adenocarcinoma Squamous cell carcinoma Not proven	10 (37.0%) 6 (22.2%) 11 (40.7%)
Tumor location Upper lobe Middle lobe Lower lobe	9 (33.3%) 1 (3.7%) 17 (63.0%)
Clinical target volume, cc (range) < 47.5 ≥ 47.5	Median 53.4 (19.8 – 321.8) 11 (40.7%) 16 (59.6%)
Breath control Free breathing Gating or BH	17 (63.0%) 10 (37.0%)

ILD, interstitial lung disease; IPF, idiopathic pulmonary fibrosis; CPFE, combined pulmonary fibrosis and emphysema; COPD, chronic obstructive pulmonary disease; BH, breath holding technique.

Although the study protocol did not impose any restrictions, all enrolled patients in this study were observed to have received no additional systemic therapy, including target therapy or immunotherapy, until the disease progression after RT.

### Local control and survival

**Figure 1:**
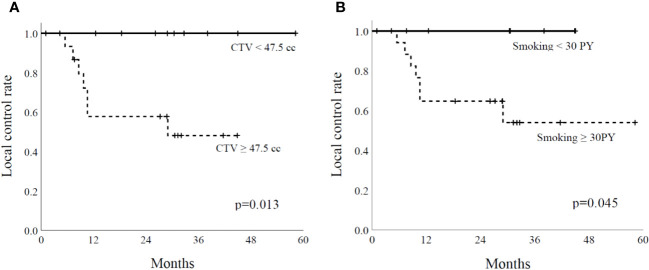
Local control rate with clinical target volume (CTV) **(A)** and smoking **(B)**. PY, pack-year.

The median follow-up period was 28.9 months. The 2-year local control rate was 73.5%. In univariate analysis (**Table 2**), smoking history indicated significantly different outcomes, with a 2-year local control rate of 100% for patients with a smoking history < 30 pack-years and 64.7% for those ≥ 30 pack-years (p=0.045) (**Figure 1A**). Furthermore, CTV demonstrated a substantial impact, with local control rates of 100% for tumors < 47.5 cc and 57.8% for tumors ≥ 47.5 cc (p=0.013) (**Figure 1B**). The two-year OS rate was 76.5%, and no significant factors were observed in the univariate analysis.

**Table 2:** Local control and survival analysis.

Characteristics	Local control		Overall survival	
	2-year	p-value	2-year	p-value
Age (years) < 75 ≥ 75	69.2% 88.3%	0.893	71.4% 83.1%	0.584
Smoking < 30 ≥ 30	100% 64.7%	0.045	85.7% 72.7%	0.470
Underlying lung disease Non-ILD ILD (IPF or CPFE)	85.7% 60.0%	0.175	87.1% 60.6%	0.134
Clinical T-stage T1 T2-3	85.7% 51.4%	0.062	85.7% 65.8%	0.189
Histology Adenocarcinoma Squamous cell carcinoma Not proven	75.0% 66.7% 80.0%	0.389	78.8% 100% 63.6%	0.295
Tumor location Upper or middle lobe Lower lobe	70.0% 79.0%	0.982	80.0% 74.7%	0.751
Clinical target volume < 47.5 cc ≥ 47.5 cc	100% 57.8%	0.013	80.0% 74.7%	0.665
Breath control Free breathing Gating or BH	71.4% 66.7%	0.618	80.7% 70.0%	0.528

ILD, interstitial lung disease; IPF, idiopathic pulmonary fibrosis; CPFE, combined pulmonary fibrosis and emphysema; BH, breath holding technique.

### Radiation-related toxicity and change(s) in lung function

Grade ≥ 2 RT-related toxicities included grade-2 radiation pneumonitis in three (11.1%) patients and grade 3 radiation pneumonitis in 1 patient (3.7%), of whom two patients had underlying idiopathic pulmonary fibrosis. Grade 3 radiation dermatitis occurred in 1 (3.7%) patient. No grade 4 or 5 toxicities were observed.

As an indicator of lung function, the mean initial FEV_1_ was 1.85 L, which did not change significantly until 22 months after RT. However, the diffusing capacity of the lungs for carbon monoxide (DLCO) decreased slightly from 60.1% to 56.2% 1 month after RT (p=0.014). At 4–10 months, the changes were not significant, whereas a significant decrease was observed at 13 and 19–22 months after RT (**Supplementary Table 2**).

### QoL

**Figure 2:**
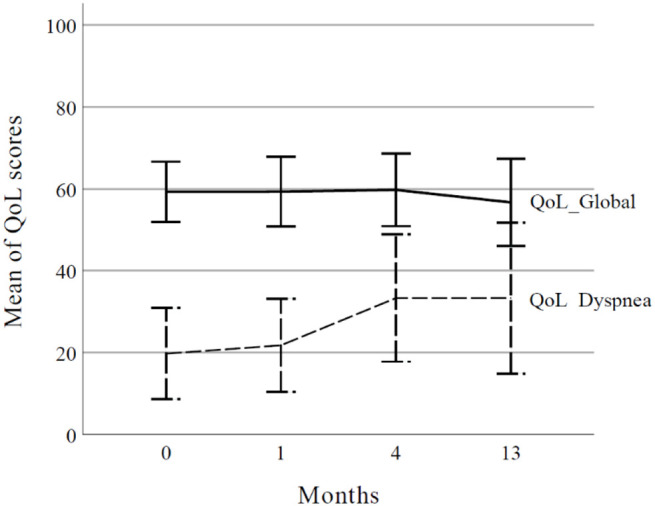
Global health status and dyspnea score according to the European Organization for Research and Treatment of Cancer Quality of Life Core 30 (EORTC QLQ-C30). The curve represents mean values and error bars represent the 95% confidence interval. QoL, quality of life.

According to EORTC-QLQ-C30 results, the global score did not change until 13 months after RT (**Supplementary Table 3**). However, dyspnea worsened, starting at 4 months after RT (**Figure 2**, p=0.047) and was maintained for 13 months after RT (p=0.028). Among other factors, the physical score improved 13 months after RT (p=0.015), and the constipation score decreased at 4 months (p=0.021).

## Discussion

This study investigated the outcomes of hypofractionated RT with PBT as a potential alternative treatment in patients with medically inoperable conditions. Consistent with previous studies, hypofractionation yielded promising results in terms of local control. Notably, for tumors with a smaller CTV, excellent local control rates were observed; however, tumors with a larger CTV exhibited relatively lower local control rates. Adverse effects were manageable and, despite a mild worsening of dyspnea, no severe changes in patient QoL were observed during follow-up.

Hypofractionation constitutes a broader framework that includes both conventional fractionation and SBRT. Unlike the well-established outcomes of SBRT, there is a lack of consensus regarding the optimal dosage and frequency of hypofractionation, particularly in challenging situations. The American Society for Radiation Oncology recommended considering 6–15 fractions of hypofractionated RT or conventional fractionated RT for central lung tumors (9). When determining such a dose, it is relevant to consider that a biologically effective dose (BED) of ≥ 100 is recommended for local control of lung cancer (18, 19). Given that a two-year local control rate of approximately 80–96% has been reported for 54–60 Gy in 3–5 fractions (BED 132–151.2 Gy_10_) and–60–75% for 60 Gy in 15–20 fractions (BED 78–84 Gy_10_), the chosen dose of 64 Gy in 8 fractions (BED 115.2 Gy_10_) in our study appears to be a strategic choice that aims to maximize local control while minimizing SBRT-associated side effects (3, 5, 7).

In our study, lower levels control was associated with larger tumors. Similarly, a lower local control rate was observed using SBRT in large tumors. Some studies have reported a local control rate of approximately 80–85% in tumors ≥ 3–5 cm (20, 21). Adjusting the radiation dose to enhance local control of larger tumors may be one approach; however, advocating for further hypofractionated RT in patients in whom SBRT is challenging could prove to be difficult. Therefore, additional systemic therapies may be viable options. However, a National Cancer Database analysis reported that adjuvant chemotherapy increased overall mortality (22). Recent studies have actively explored the combination of immunotherapy and hypofractionated RT (23). Thus, as a potential follow-up to our study—combination immunotherapies—could be a worthy avenue of exploration.

Patients who undergo hypofractionated RT frequently exhibit either temporary or permanently reduced lung function (24). In addition, dyspnea worsening due to radiation pneumonitis, which often occurs between 3 and 6 months post-treatment, was reported to have a broad range of 17%–66% (25). PBT is expected to have fewer side effects, owing to its ability to limit the radiation dose to the lungs. In our study, we observed a decrease in DLCO and concomitant deterioration in dyspnea-related QoL. However, despite these observations, the dyspnea score remained at a relatively manageable level, the global score exhibited no change, and the physical score even showed an increased. Given these findings, the impact of PBT on patient QoL may not be substantial. Considering the low level of observed toxicity, it can be inferred that the RT regimen used in our study appears to be sufficiently safe for application, even in inoperable patients.

Our study had several limitations. First, it used a single-arm design without a control group, for which we could not compare the results between PBT and photon therapy. Second, based on a historical local control rate of 60% with conventional RT, the observed local control rate in this study was 73.5%, and the sample size was calculated assuming a local control rate of 85%. Therefore, the sample size in this study may have been insufficient, and we were unable to conduct a significant multivariate analysis. Despite closely aligning with existing research findings, nevertheless, our study revealed a notable advantage of hypofractionated PBT, characterized by minimal grade 3 or higher toxicities, manageable patient-reported dyspnea, and the preservation of QoL. Future research should prioritize larger, well-controlled studies to better determine the true potential and comparative effectiveness of the treatment.

## Conclusion

This study evaluated the effectiveness of moderately hypofractionated PBT in patients who were medically unfit for surgery. The results demonstrated promising outcomes in terms of local control, especially for smaller tumors, whereas control rates in larger tumors were relatively lower. Despite manageable side effects and a mild increase in dyspnea, there were no significant changes in patient QoL. As such, results of this investigation emphasize the need for larger, well-controlled studies to better understand the treatment potential and to continue efforts to find improved therapeutic strategies.

## Data availability statement

The raw data supporting the conclusions of this article will be made available by the authors, without undue reservation.

## Ethics statement

The studies involving humans were approved by Institutional Review Board of Samsung Medical Center. The studies were conducted in accordance with the local legislation and institutional requirements. The participants provided their written informed consent to participate in this study.

## Author contributions

KY: Formal analysis, Investigation, Methodology, Visualization, Writing – original draft. JN: Conceptualization, Investigation, Validation, Writing – review & editing. HP: Resources, Writing – review & editing. HY: Resources, Writing – review & editing. SS: Resources, Writing – review & editing. HP: Conceptualization, Resources, Supervision, Writing – review & editing.
