# Supplementary material for: Prospective study investigating hypofractionated proton beam therapy in patients with inoperable early stage non-small cell lung cancer
Source: Front Oncol. 2024 Feb 20;14:1296172. doi: 10.3389/fonc.2024.1296172 (PMC10912465; doi:10.3389/fonc.2024.1296172)
Supplement: Supplementary file 1 [file Table_1.docx]

**Supplementary table 1** Dose-volume parameters in the organs-at-risks.

| **Parameters^*^** | **Mean (range)** |
| --- | --- |
| Total lung  Mean dose (cGy)  V_5_ (%)  V_20_ (%) | 436 (130 – 826)  12.6 (4.6 – 22.4)  8.0 (2.5 – 15.2) |
| Heart  V_40_ (%) | 5.0 (0 – 40%) |
| Esophagus  D_max_ (cGy)  D_mean_ (cGy) | 824 (0 – 1791)  20 (0 – 58) |
| Spinal cord  D_max_ (cGy) | 626 (0 – 1953) |

*^*^V_D_* percentage volume of organ receiving ≥ D Gy.

**Supplementary table 2** Pulmonary-function test.

| Parameter | FEV1 (L) | | DLCO (%) | |
| --- | --- | --- | --- | --- |
|  | mean | p-value^*^ | mean | p-value^*^ |
| Baseline | 2.01 |  | 60.12 |  |
| 1 month | 2.00 | 0.778 | 56.23 | 0.014 |
| 4 months | 1.91 | 0.204 | 56.30 | 0.062 |
| 7 months | 1.80 | 0.193 | 58.30 | 0.075 |
| 10 months | 1.80 | 0.221 | 54.77 | 0.102 |
| 13 months | 1.88 | 0.666 | 55.31 | 0.002 |
| 16 months | 1.74 | 0.293 | 53.41 | 0.129 |
| 19 months | 1.52 | 0.167 | 44.57 | 0.045 |
| 22months | 1.28 | 0.115 | 43.29 | 0.029 |

^*^Paired t-test comparing mean values at the time before RT and specific months after RT.

**Supplementary table 3** The results of EORTC-QLQ-C30.

|  | | Baseline | 1 month | | 4 months | | 13 months | |
| --- | --- | --- | --- | --- | --- | --- | --- | --- |
|  |  | mean | mean | p-value^*^ | mean | p-value | mean | p-value^*^ |
| Global health status | | 59.3 | 59.3 | 0.935 | 59.7 | 0.934 | 56.7 | 0.312 |
| Functional status | Physical | 76.0 | 72.3 | 0.264 | 70.6 | 0.239 | 66.2 | 0.015 |
|  | Role | 82.7 | 81.4 | 0.796 | 81.9 | 0.781 | 73.3 | 0.153 |
|  | Emotional | 80.5 | 82.0 | 0.658 | 75.4 | 0.318 | 90.6 | 0.108 |
|  | Cognitive | 81.5 | 75.6 | 0.223 | 80.6 | 0.477 | 82.2 | 0.264 |
|  | Social | 84.0 | 80.8 | 0.513 | 86.1 | 0.910 | 92.2 | 0.136 |
| Symptom | Fatigue | 21.4 | 24.3 | 0.546 | 31.5 | 0.156 | 31.8 | 0.181 |
|  | Nausea / Vomiting | 3.5 | 4.5 | 0.713 | 4.2 | 0.908 | 14.4 | 0.220 |
|  | Pain | 8.0 | 11.5 | 0.232 | 12.5 | 0.338 | 14.5 | 0.357 |
|  | Dyspnea | 19.8 | 21.8 | 0.770 | 33.3 | 0.047 | 33.3 | 0.028 |
|  | Insomnia | 19.7 | 25.6 | 0.381 | 33.3 | 0.161 | 24.4 | 0.363 |
|  | Appetite loss | 18.5 | 12.8 | 0.448 | 22.2 | 0.266 | 24.4 | 0.090 |
|  | Constipation | 13.6 | 20.5 | 0.136 | 18.1 | 0.021 | 11.1 | 0.999 |
|  | Diarrhea | 4.9 | 9.0 | 0.185 | 11.1 | 0.213 | 11.1 | 0.334 |
| Financial difficulties | | 9.8 | 9.0 | 0.259 | 13.9 | 0.110 | 4.4 | 0.999 |

^*^Paired t-test comparing mean values at the time before RT and specific months after RT
